# Long-Term Outcome Following Concomitant Surgical Ablation for Atrial Fibrillation at University Hospital Basel: A Retrospective Study

**DOI:** 10.3390/medicina61010041

**Published:** 2024-12-30

**Authors:** Elodie Hersperger, Nicolas Schaerli, Brigitta Gahl, Oliver Reuthebuch, Friedrich S. Eckstein, Bernhard Winkler, Martin T. R. Grapow

**Affiliations:** 1 University Heart Center, University Hospital Basel, 4031 Basel, Switzerland; 2 Herzchirurgie Aarau, Hirslanden Medical Center, 5000 Aarau, Switzerland; 3 Department of Cardiovascular Surgery KFL, Vienna Health Network, 1210 Vienna, Austria; 4 Heart Center Hirslanden Zurich, 8008 Zurich, Switzerland

**Keywords:** atrial fibrillation, surgical ablation, cardiac surgery

## Abstract

*Background and Objectives*: This study aims to examine the success of concomitant surgical ablation in patients with atrial fibrillation after one, three, and five years. Additionally, important predictors for rhythm outcome and rates of permanent pacemaker implantations were analyzed. *Materials and Methods*: In this retrospective study, we included patients who were referred to the University Hospital of Basel, Switzerland, between 2011 and 2017. Primary outcome was one-year success of surgical ablation. Secondary outcomes include heart rhythm during entire follow-up, three- and five-years success, and need for additional arrhythmia interventions (incl. pacemaker implantation). *Results*: A total of 120 patients were analyzed and divided into two groups: pulmonary vein isolation (*n* = 55) and left atrial (*n* = 65). The median follow-up time was 4.4 years. Sinus rhythm was present in 66%, 64%, and 67% after one, three, and five years, respectively. When adjusted for preoperative type of atrial fibrillation, left atrial lesion set increased the chance of achieving sinus rhythm within 5 years by factor 6.5. The pacemaker-implantation rate was 22%, with significantly more pacemaker implantations in the left atrial group (9% vs. 32%, *p* = 0.0043). *Conclusions*: These real-world data demonstrate the high success rate of concomitant surgical ablation for atrial fibrillation. Our study highlights the importance of preoperative discussion in an interdisciplinary heart team to weigh the effectiveness of surgical ablation against the risk of a pacemaker implantation.

## 1. Introduction

Atrial fibrillation (AF) is the most common sustained cardiac arrhythmia, affecting over 43.6 million individuals globally (as of 2016). The lifetime risk of developing AF at an index age of 55 years is 37% for individuals of European ancestry. AF is associated with an increased risk of various complications, such as heart failure, stroke, sudden death, cognitive decline, depression, and all-cause mortality. As a result, AF represents a significant burden on both patients and healthcare systems, independently increasing the risk of all-cause mortality by 1.5-fold in men and 2-fold in women. The clinical presentation of AF can range from asymptomatic (50–87%) to severely symptomatic, including hemodynamic instability. Common symptoms include palpitations, dyspnea, and fatigue. AF can be diagnosed through a thorough assessment of medical history and AF-related symptoms, with confirmation by electrocardiogram [1,2].

Surgical ablation emerged as a treatment modality for AF thirty years ago and has since been modified and improved. The treatment of AF is complex and involves anticoagulation, rate control, and rhythm control. Several treatment modalities are available, including drugs, catheter based pulmonary vein ablation (PVI), cardioversion, and surgical ablation, which is the focus of this study. The concept of surgical ablation is to create lesions of conduction block in a maze pattern to prevent the generation and propagation of macro-re-entry circuits in the atria, while maintaining the atrial contraction [3,4]. The effects of surgical ablation on freedom from AF one-year post-procedure have been clearly demonstrated [2]. The American Association for Thoracic Surgery states that freedom from AF after one year should be 70% or greater [5]. Concomitant surgical ablation has demonstrated to be a safe procedure when added to routine cardiac surgical setting without increasing morbidity and mortality in patients [1,5,6]. However, there is evidence that surgical ablation increases the need for pacemaker implantation postoperatively [7,8]. In their recent guidelines, the European Society of Cardiology (ESC) recommends considering concomitant AF ablation in all patients with a history of AF undergoing cardiac surgery (Class of recommendation IIa, level of evidence A) [2].

Most surgical ablation studies do not report outcomes exceeding a two-year follow-up. In the past years, interest in studies investigating long-term effects of surgical ablation has grown. Therefore, this retrospective single-center study aims to examine the success rate of concomitant surgical ablation in patients with AF with an up to eight year follow-up period. Moreover, the focus of this study was to identify variables that predict freedom from AF, such as the preoperative type of AF, lesion set performed, energy source used, and patient characteristics. Finally, we aimed to analyze the need for permanent pacemaker implantation.

## 2. Materials and Methods

*Patients and study design:* This retrospective single-center study included patients who underwent concomitant surgical ablation for AF between January 2011 and January 2017 at the University Hospital of Basel, Switzerland. The study population consisted of patients with paroxysmal, persistent, and long-standing persistent AF as defined by the 2020 ESC-Guidelines [2]. The inclusion criterion was surgical ablation performed concomitant to cardiac surgery addressing valvular or ischemic heart disease. Exclusion criteria were stand-alone procedure, surgical ablation for atrial flutter (AFL), and intraoperative death. Patient data used in this study were obtained from the electronic medical records of University Hospital of Basel. Since follow-up after surgical ablation was mostly performed by peripheral cardiologists, all rhythm status and medication after discharge were obtained by contacting the patient’s treating cardiologist or primary care physician. We included follow-ups performed from the date of surgical ablation until October 2019. We evaluated the heart rhythm in available 12-chanel electrocardiograms (ECG), Holter monitorings, and/or cardiac implantable electronic device (CIED) interrogations. If a patient showed no atrial arrhythmia on 12-channel ECG, but Holter monitoring or CIED detected AF or AFL, rhythm status was defined according to the Holter monitoring or CIED. At each follow-up, current medication status was collected.

*Outcome and definitions:* The primary outcome was one-year success of surgical ablation according to 2017 Heart Rhythm Society consensus statement [9]. One-year success was defined as freedom from AF or AFL between 6 and 18 months after the ablation procedure. We defined recurrence of AF or AFL as a supraventricular arrhythmia that was documented in a 12-lead ECG or was otherwise recorded to last for at least 30 s on Holter monitoring. In patients having a CIED with an atrial lead, the treating cardiologist’s analysis of tracing storage was used. Secondary outcomes were heart rhythm during entire follow-up, three-years and five-years success of surgical ablation (assessment closest to 3 and 5 years within +/−6 months), need for additional arrhythmia interventions (repeat ablation procedures, electrical cardioversion), pacemaker, use of oral anticoagulation (OAC), use of antiarrhythmic drugs therapy (AAD), perioperative morbidity, and mortality.

*Intervention and ablation methods*: All patients underwent surgical ablation in combination with other cardiac procedures. Main procedures were valve surgery (repair or replacement of aortic, mitral, and tricuspid valve) and/or coronary artery bypass grafting (CABG). Surgical ablation procedures for AF were divided into three different groups according to the Heart Rhythm Society expert consensus 2017 [9]: (1) PVI alone (PVI group); (2) PVI combined with left atrial (LA) lesion sets; (3) and biatrial Cox–Maze procedure. For the analysis of this study, the biatrial and left atrial groups were taken together (LA group). The choice of lesion set was left to the discretion of the surgeon, taking into consideration the type of AF and type of surgery. In general, when surgery did not require opening of the left atrium, only PVI was performed. When concomitant surgery included opening of the left atrium, LA or BA lesion set was applied, especially in patients with persistent and long persistent AF. Ablation in the PVI group was mostly performed with a bipolar radiofrequency clamp. Patients who underwent LA lesion set had a line from the incision from the box-lesion to the P3-part of the mitral annulus in addition to PVI. Moreover, these patients mostly received a line to the rim of the left atrial appendage (LAA). Patients in the BA group had heterogeneous ablation sets used, all of which include lines in both atria. When LAA occlusion was performed, excision, ligation, and clipping were used. Surgical ablation lines were made using cryothermal energy and mono- and bipolar radiofrequency. The choice of energy source was made according to surgeons’ preference. Conduction block created by surgical ablation was not verified intraoperatively.

*Postsurgical treatment:* Most patients received postoperative OAC with Phenprocoumon (Marcoumar^®^, Viatris Pharma GmBH, 6312 Steinhausen, Switzerland) with a target international normalized ratio of 2.0–3.0. Continuation of OAC was recommended for at least 3 months, then withdrawal was left to the discretion of the treating cardiologist. Necessity for postoperative conversion was decided on an individual basis and was performed by Amiodarone (Cordarone^®^, Sanofi-Aventis SA, 1214 Vernier, Switzerland) or electric cardioversion. Reasons for AAD at discharge were postoperative AF/AFL or continuation from preoperative AAD therapy. The choice for prolonging the use of AADs or additional antiarrhythmic intervention (catheter ablation, repeated cardioversion) was an individual decision taken by the treating cardiologist. Follow-up visits with evaluation of OAC and AAD therapy was minimally recommended after 3 and 12 months. The future timing of outpatient visits and antiarrhythmic drugs strategy was left to the discretion of the treating cardiologist.

*Statistical analysis:* The study cohort was selected based on our prospectively maintained clinical registry. We checked the registry for completeness monthly using the operating room planning tool as reference and for consistency according to a scheme we developed. After selecting all patients who underwent ablation from the registry, we collected follow-up data of those who had consented. We did not conduct a formal sample size calculation. We applied the conventional statistical cut-offs, e.g., confidence intervals are based on 1.96 × standard errors and *p* values < 0.05 are considered statistically significant. To evaluate primary outcome, we included the measurement closest to one-year post-ablation in patients having undergone heart rhythm assessment between 6 and 18 months and used logistic regression to derive odds ratios of sinus rhythm (SR) and type of ablation. We found that SR during follow-up was strongly associated with type of preoperative AF, so we included it as an ordered category in the model. We used the same approach for SR at three and five years. To investigate whether more extensive surgical ablation is associated with longer postoperative SR periods, we used multilevel logistic models, including SR within 5 years of follow-up as dependent variable and time since ablation and type of preoperative AF, as fixed factors and patient as a random factor. SR or AF during follow-up was assumed to last from diagnosis until diagnosis of the opposite. We summarized continuous variables as median and interquartile range and tested for difference between treatment groups using Wilcoxon rank-sum test. We showed categorical variables as number with percentage and checked for differences using Fisher’s exact test. All analyses were carried out using Stata 16 (Stata Corp., College Station, TX, USA); the line plot was created in R (The R project, V).

## 3. Results

### 3.1. Baseline Characteristics

Between January 2011 and January 2017, a total of 127 patients underwent cardiac surgery with concomitant ablation at the University Hospital of Basel. Seven patients were excluded from the current analysis: one patient who underwent stand-alone procedure, five patients who had surgical ablation for AFL, and one patient who died intraoperatively. The 120 patients analyzed were divided in PVI group (*n* = 55) and LA group (*n* = 65). The LA group included four patients who had a BA lesion set.

The baseline characteristics, including the accompanying diseases, are presented in Table 1. Both groups were similar regarding demographic characteristics and comorbidities; mean age was 71 (66 to 75). Regarding preoperative type of AF, 50% of the patients had paroxysmal AF while 24% had persistent and 26% had long-standing persistent AF. There were significantly more patients with paroxysmal AF in the PVI group (*p*-value < 0.001). There was a tendency towards more CABG and mitral valve repair/replacement in the LA group, whereas there were significantly more aortic valve repair/replacement in the PVI group (*p*-value < 0.001). LAA occlusion was performed in 59% of the patients, mostly by ligation. The energy source used was mostly radiofrequency (bipolar 48% and monopolar 29%). Cryothermal energy was used in 23% of the patients, particularly in the LA group. The median follow-up time was 4.4 years (max 8 years). Rhythm assessment was conducted in 99% by resting ECG.

### 3.2. Rhythm Outcome

SR was present in 63 of 95 patients (66%) after one year, in 43 of 67 patients (64%) after three years, and 25 of 37 patients (67%) after five years. The chance of maintaining SR in patients with paroxysmal AF preoperatively was 82.6%, 81.8%, and 82.4% after one, three, and five years, respectively. The chance of maintaining SR in patients with persistent AF preoperatively was 72.7%, 71.4%, and 100% after one, three, and five years, respectively. The chance of maintaining SR in patients with long-standing AF preoperatively was 33.3%, 30.0%, and 18.2% after one, three, and five years, respectively (Table 2). We did not find a crude association between type of ablation (PVI group vs. LA group) and SR at one, three, or five years after surgical ablation (Table 3). When analyzing SR adjusted for preoperative type of AF within five years of follow-up, LA lesion set compared to PVI increased the chance of achieving SR by factor 6.5 (95% CI [1.78 to 43.0], *p* < 0.001).

Clinically relevant parameters with significant association with freedom from AF within 5 years were patient’s age (*p*-value 0.016) and LA size (*p*-value 0.027). The chosen energy source did not have a significant influence on achieving SR (Table 4).

### 3.3. Cardioversion and Catheter Ablation

Nineteen patients underwent at least one ECV after surgical ablation, eleven of these patients had their first ECV within the first three months after surgical ablation. Five patients (9%) in PVI group underwent cardioversion compared to fourteen patients (22%) in LA group. Five patients underwent catheter ablation in the follow-up, all of whom were from the LA group. These patients received a total of eight ablations: two cavo-tricuspid isthmus ablations due to typical AFL (not related to the surgery), two mitral isthmus dependent flutter after mitral isthmus ablation during surgery, and three cases with different (scar related), multiple tachycardias. One of the latter could not be successfully ablated, and therefore AV node ablation was performed.

### 3.4. Pacemaker Implantation

Overall, 26 of 120 patients (22%) needed a pacemaker implantation, and 46% were implanted in hospital (Table 5). The main reasons for pacemaker implantations were sinus node disturbance (34%), advanced atrioventricular block (38%), intraventricular block (8%), or combined blocks. The median time between surgery and pacemaker implantation was 352 days, and 505 days between surgery and late-onset pacemaker implantation. There were significantly more pacemaker implantations in the LA group than in the PVI group (*p* = 0.0043). When analyzing subgroups, all four patients with BA lesion set had a pacemaker implantation. There was no significant difference between energy sources and pacemaker implantation rates (*p* = 0.061).

### 3.5. Use of Oral Anticoagulation and Antiarrhythmic Drug Therapy

OAC (mostly Phenprocoumon) was used in 75% of the patients preoperatively and in 83% of the patients postoperatively. Discontinuation of OAC was conducted mostly in the year after ablation. AAD was given to 16% of the patients preoperatively and to 28% of the patients postoperatively. Amiodarone was the most frequently used AAD. The use of AAD decreased to 12% one-year post-ablation.

### 3.6. Morbidity and Mortality

The most common postoperative morbidities were pericardial and pleural effusion. There was no reoperation for bleeding, though one patient got a wound infection. Twenty-three patients died during the follow-up period. Neither the mortality nor the postoperative morbidities were significantly different between the PVI and LA group.

**Figure 1:**
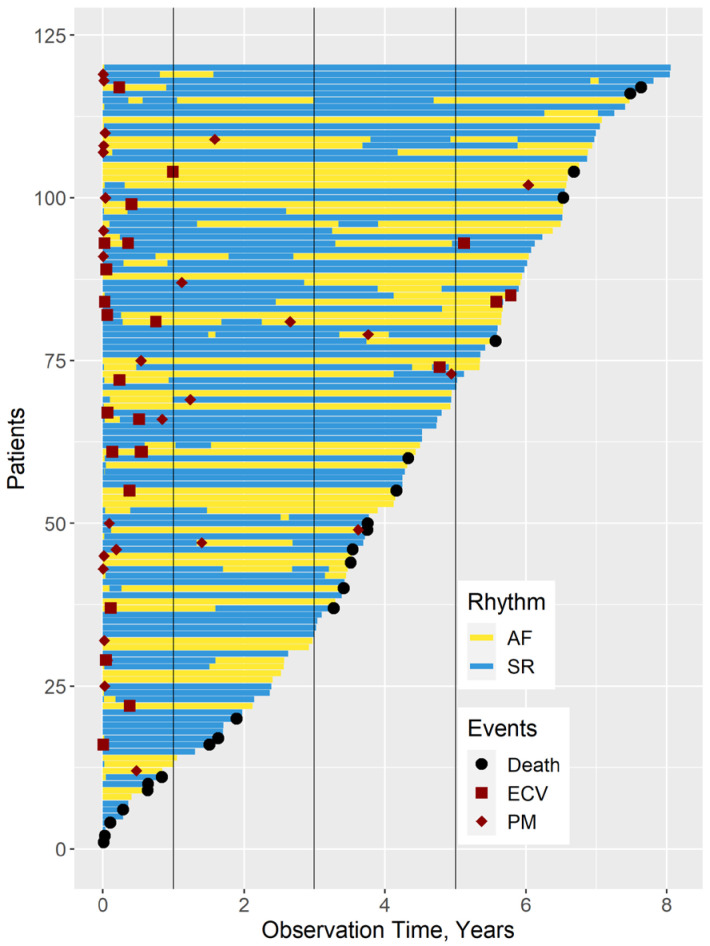
Overview of the results.

Figure 1 gives an overview of all results in form of a line plot.

## 4. Discussion

We report four major findings in this study. First, overall SR was present in 63 of 95 patients (66%) one-year post-ablation, in 43 of 67 patients (64%) three-years post-ablation, and 25 of 37 patients (67%) five-years post-ablation. Second, in patients with paroxysmal, persistent, and long standing-persistent AF, SR was present in 83%, 73%, and 33%, respectively one-year post-ablation. These numbers remained stable after three and five years. Third, LA lesion set compared to PVI, when adjusted for preoperative type of AF, significantly increased the chance of achieving SR by factor 6.5. Fourth, the rate of pacemaker implantations was 22% with higher prevalence in the LA group.

*Rhythm outcome:* The success rates reported in our study one-year post-ablation are in accordance with the results of current reports analyzing surgical ablation in patients with AF [6,10,11,12]. In the last years, researchers have become increasingly aware of the importance of longer follow-up periods to assess the outcome of surgical ablation. Our follow-up period with a median of 4.4 years is therefore a major advantage of our study. Our results suggest a long-term effect of surgical ablation on rhythm outcome. These findings are consistent with the results of the prospective study by Ad et. al. [13].

ESC-Guidelines [2] state that the most effective ablation treatment for AF is lesion sets where lines exceeding the isolation of pulmonary veins are performed. Indeed, our study showed when adjusted to type of AF (including all assessments within 5 years post-ablation), the LA lesion set, compared to PVI, increased the chance of achieving SR. In most centers, the choice of lesion set is made according to the type of AF and the concomitant types of cardiac surgery. PVI is the common lesion set in patients with paroxysmal AF, except when concomitant surgery includes opening the left atrium. Our data suggest that LA lesion set could be considered as primary treatment in patients with paroxysmal AF to improve the rhythm outcome. This contradicts with the findings of Gilinov et al. [14], where 152 patients undergoing combined surgical ablation of paroxysmal AF and mitral valve disease were retrospectively analyzed. Gilinov et al. concluded that PVI alone could be an adequate treatment for patients with paroxysmal AF, particularly of short duration. Further randomized-controlled-trials specifically analyzing paroxysmal AF and type of ablation are needed to assess the benefit of lesions exceeding PVI in these patients. Regarding persistent AF, several studies showed that BA lesion is more effective than left-sided only [2]. This could not be commented on in our study because of the small sample size of patients with a BA lesion set.

Surprisingly, in our study, the choice of energy source had no impact on the rhythm outcome. Most evidence of energy sources used to perform surgical ablation are found for bipolar radiofrequency clamps and cryothermy [2]. Monopolar radiofrequency is no longer recommended for surgical ablation due to its inefficient transmural lesions [5]. However, in our study, monopolar radiofrequency was still in use and was mostly applicated in patients with LA lesion set. Therefore, the increased success of the LA lesion set after adjustment for type of AF is even more surprising.

*Pacemaker implantation:* Surgical ablation is known to increase the need for pacemaker implantation postoperatively compared to patients treated without surgical ablation [7,8]. In our study, the rate of pacemaker implantations was 22% and thus is in agreement with the rates reported by ESC-Guidelines (from 6.8% to 21.5%) [2]. Our study showed a significantly higher proportion of pacemaker implantation in the LA lesion group compared to PVI group. When analyzing the subgroup of patients with a BA lesion set who are included in the LA lesion group, all four of the patients had a pacemaker implantation after surgical ablation. This is in accordance with recent studies that identified right-sided lesions to be the main reasons for pacemaker implantations [1,2,15,16,17]. A possible explanation is that additional right atrial lesions might damage the sinus node or atrioventricular node and its blood supply. Furthermore, surgical ablation might unmask preexisting disease of the sinus or atrioventricular node in patients with persistent or long-standing persistent AF [16]. It must be kept in mind that concomitant multivalve surgery is also an important risk factor for postoperative atrioventricular block [8]. In our study, 95 patients (80%) had valve surgery, which could explain the high rate of pacemaker implantations. Kakuta et al. identified longer preoperative AF duration and older age as risk factors for late-onset pacemaker implantation after modified Cryo-Maze procedure [18]. However, they showed no difference in survival, cumulative incidence of cerebrovascular accidents and restoration of SR between patients with and without pacemaker implantation. Further studies are needed to confirm these results. Currently, these findings should be considered when evaluating older patients with long-standing persistent AF for surgical ablation.

*Repeated procedures:* In our study, five patients received at least one catheter ablation to treat atrial arrhythmias that were related to the surgical ablation. Four of these patients could be treated successfully with catheter ablation. One patient had multiple arrhythmias that could not been successfully ablated and therefore an AV node ablation was performed. The mechanism of the arrhythmia was either persistent conduction through a region that was targeted at concomitant surgical ablation or a gap between a surgical incision and an anatomic boundary serving as a channel for a regular tachycardia. This is in line with previous studies [19]. In our study, only 4% of the patients required catheter ablation. This is a small percentage compared to other studies reporting this endpoint and underlines the success of surgical ablation in our study [20].

Recent studies have highlighted the emerging role of newer drugs, such as sodium-glucose cotransporter 2 inhibitors and Glucagon-like Peptide 1 receptor agonists, in reducing the risk of AF recurrence [21,22]. However, these pharmacological agents were not used in our patient cohort, as they were not standard of care during the study period. Further research is needed to explore the role of these drugs in reducing the risk of AF recurrence.

## 5. Limitations

The main limitation of this study is the non-randomized retrospective study design, which cannot avoid confounders and selection and detection bias. Since the follow-up was not pre-specified, the intervals of the collected data varied, and the number of patients lost to follow-up was high. Since AF occurs and returns repeatedly, possible recurrences could have been missed due to irregular follow-up and could lead to overestimation of the success rate of surgical ablation. On the other hand, it is probable that patients in sinus rhythm are more likely to be lost to follow-up than patients with symptomatic arrhythmias because asymptomatic patients might visit their treating physician less often. This could lead to an underestimation of the success rate of concomitant surgical ablation. However, monitoring of patients was mostly conducted using 12-channel ECG, leading to a potential overestimation of the success rate. Ad et al. showed that compared to long-term monitoring, 12-channel ECG overestimates the success rate after surgical ablation by 12% [23]. This study focused on rhythm outcome and was not designed to analyze patient-important outcomes such as stroke, heart failure, and health-related quality of life. Effects on these endpoints are not well established and should be integrated into study-design of future trials about surgical ablation. Finally, the cohort contained a variety of preoperative types of AF and concomitant surgery. However, this mixed population represents the typical clinical population and therefore allows clinical application.

## 6. Conclusions

The presented real-world data in this study demonstrate the high success rate of concomitant surgical ablation for AF. This emphasizes the role of surgical ablation as a treatment modality for AF. Furthermore, our study showed the importance of preoperative type of AF and the role of more extended lesion sets as predictors to increase the chance of achieving SR. Nevertheless, this study demonstrated a high rate of pacemaker implantation, especially after more extended lesion sets. This highlights the importance of preoperative discussion regarding concomitant surgical ablation, preferably in an interdisciplinary Heart Team consisting of cardiac surgeons and cardiac electrophysiologists, to weigh the effectiveness of ablation against the potential risk of a pacemaker implantation.

## Figures and Tables

**Table 1:** Baseline characteristics.

	N	Total (N = 120)	Pulmonary Vein Isolation (N = 55)	Left Atrial Lesion Set (N = 65)	*p*-Value
*Demography*					
Female	120	38 (32%)	14 (25%)	24 (37%)	0.24
Age	120	71 (66 to 75)	72 (68 to 77)	69 (64 to 75)	0.12
*Comorbidities*					
Diabetes mellitus	120	9 (7.5%)	6 (11%)	3 (4.6%)	0.30
Hypertension	120	96 (80%)	47 (85%)	49 (75%)	0.25
Hypercholesteremia	120	67 (56%)	35 (64%)	32 (49%)	0.14
Current smoker	120	9 (7.5%)	2 (3.6%)	7 (11%)	0.18
Peripheral artery disease	120	9 (7.5%)	5 (9.1%)	4 (6.2%)	0.73
History of stroke	120	14 (12%)	8 (15%)	6 (9.2%)	0.40
Prior myocardial infarction	120	30 (25%)	17 (31%)	13 (20%)	0.21
Renal disease	120	2 (1.7%)	1 (1.8%)	1 (1.5%)	1.00
*Atrial fibrillation*					
Type	120				<0.001
Paroxysmal		60 (50%)	40 (73%)	20 (31%)	
Persistent		29 (24%)	6 (11%)	23 (35%)	
Long-standing persistent		31 (26%)	9 (16%)	22 (34%)	
Duration	118				0.76
<3 months		22 (18%)	11 (20%)	11 (17%)	
3–6 months		28 (23%)	13 (24%)	15 (23%)	
6–12 months		16 (13%)	6 (11%)	10 (15%)	
12–24 months		14 (12%)	8 (15%)	6 (9.2%)	
>24 months		38 (32%)	15 (27%)	23 (35%)	
*Echocardiography*					
Left atrium enlarged	90	85 (71%)	36 (65%)	49 (75%)	0.31
Ejection fraction (%)	120	56 (50 to 63)	55 (45 to 60)	60 (50 to 65)	0.027
*Surgical data*					
Procedure groups	120				<0.001
CABG		24 (20%)	19 (35%)	5 (8%)	
CABG and valve(s)		40 (33%)	17 (31%)	23 (35%)	
Valve(s) and other		55 (46%)	18 (33%)	37 (57%)	
Other		1 (0.83%)	1 (1.8%)	0 (0.00%)	
Aortic valve	120	44 (37%)	32 (58%)	12 (18%)	<0.001
Mitral valve	120	57 (48%)	3 (5%)	54 (83%)	<0.001
LAA occlusion	120	71 (59%)	29 (53%)	42 (65%)	0.20
Energy source	120				<0.001
Bipolar radiofrequency		56 (47%)	53 (96%)	3 (4.6%)	
Cryothermal energy		28 (23%)	1 (1.8%)	27 (42%)	
Mono + bipolar radiofrequency		1 (0.83%)	0 (0.00%)	1 (1.5%)	
Monopolar radiofrequency		35 (29%)	1 (1.8%)	34 (52%)	

CABG: Coronary artery bypass grafting, LAA: Left atrial appendage. *p* values derived from Fisher’s exact test in categorical variables and Wilcoxon-Mann-Whitney test in continuous variables.

**Table 2:** Sinus Rhythm at 1, 3, and 5 years and preoperative type of atrial fibrillation.

Outcome	Data Available	All Patients with SR (%)	Patients with SR/All Patients with This AF Type (%)		Long-Standing Persistent
			Paroxysmal	Persistent	
One year	N = 95	63 (66%)	38/46 (82.6%)	16/22 (72.7%)	9/27 (33.3%)
Three years	N = 67	43 (64%)	27/33 (81.8%)	10/14 (71.4%)	6/20 (30.0%)
Five years	N = 37	25 (67%)	14/17 (82.4%)	9/9 (100%)	2/11 (18.2%)

AF: atrial fibrillation, SR: sinus rhythm. *p* values derived from logistic regression model.

**Table 3:** Odds ratios of pulmonary vein isolation vs. left atrial ablation and sinus rhythm at 1, 3, and 5 years and within 5 years.

Outcome	Data Available	SR Found After		OR (95% CI)	*p*-Value
		PVI	LA Lesion Set		
One year	N = 95	26	37	1.42 (0.60 to 3.35)	0.36
				* 4.34 (1.22 to 15.5)	0.024
Three years	N = 67	19	24	1.07 (0.39 to 2.92)	0.85
				* 3.59 (0.80 to 16.2)	0.097
Five years	N = 37	8	17	1.52 (0.37 to 6.29)	0.18
				* 1.38 (0.19 to 10.1)	0.75
Within five years	N = 120	126 pat. years	199 pat. years	1.42 (0.47 to 4.28)	0.54
				* 6.5 (1.78 to 43.0)	<0.001

AF: atrial fibrillation, LA: left atrial, PVI: pulmonary vein isolation, SR: sinus rhythm. * odds ratio adjusted for type of preoperative AF. *p* values derived from logistic (1, 3, 5 years) or multilevel logistic regression model (within 5 years).

**Table 4:** Predictors of postoperative freedom from atrial fibrillation.

Variable	OR (95% CI)	*p*-Value
Patient’s preoperative characteristics		
Age	0.85 (0.75 to 0.97)	0.016
Left atrium size, mm	0.83 (0.71 to 0.98)	0.027
Energy source		
Bipolar radiofrequency	Reference	
Cryothermal energy	4.61 (0.65 to 32.7)	0.13
Monopolar radiofrequency	0.17 (0.02 to 1.85)	0.15

*p* values derived from logistic regression.

**Table 5:** Pacemaker implantation.

	Total (N = 120)	PMI (N = 26)	*p*-Value
Indications			
Sinus node disturbance		9 (34%)	
Atrioventricular junction disturbance		10 (38%)	
Intraventricular conduction disturbance		2 (8%)	
Combined disturbance		5 (19%)	
Time of implantation			
In-hospital PMI		12 (46%)	
Late-onset PMI		14 (54%)	
Type of ablation			0.0043
PVI Lesion set	55	5	
LA + BA Lesion set	65	21	
Valve Repair or Replacement			
CABG		15	
CABG only		5	
Single valve		9	
Double valve		11	
Triple valve		1	
Energy Source			0.061
Monopolar radiofrequency	35	11	
Bipolar radiofrequency	56	8	
Mono + bipolar radiofrequency	1	1	
Cryothermal energy	28	6	

*p* values derived from Fisher’s exact test.

## Data Availability

The data presented in this study are available on request from the corresponding author (accurately indicate status).
